# Predictive value of computed tomography for short-term mortality in patients with acute respiratory distress syndrome: a systematic review

**DOI:** 10.1038/s41598-022-13972-x

**Published:** 2022-06-10

**Authors:** Hiroyuki Hashimoto, Shota Yamamoto, Hiroaki Nakagawa, Yoshihiro Suido, Shintaro Sato, Erina Tabata, Satoshi Okamori, Takuo Yoshida, Koichi Ando, Shigenori Yoshitake, Yohei Okada

**Affiliations:** 1 Department of Pharmacoepidemiology, Graduate School of Medicine and Public Health, Kyoto University, Kyoto, Japan; 2 Department of Radiology, Tokai University Hospital, Tokai University School of Medicine, Kanagawa, Japan; 3 Division of Respiratory Medicine, Department of Internal Medicine, Shiga University of Medical Science, Shiga, Japan; 4 Department of Respiratory Medicine, Asao General Hospital, Kanagawa, Japan; 5 Department of Respiratory Medicine, Saitama Red Cross Hospital, Saitama, Japan; 6 Department of Respiratory Medicine, Kanagawa Cardiovascular and Respiratory Centre, Kanagawa, Japan; 7 Division of Pulmonary Medicine, Department of Medicine, Keio University School of Medicine, Tokyo, Japan; 8 Department of Intensive Care Medicine, Tokyo Women’s Medical University, Tokyo, Japan; 9 Division of Respiratory Medicine and Allergology, Department of Medicine, Showa University School of Medicine, Tokyo, Japan; 10 Department of Health Science, Kyushu University of Health and Welfare, Miyazaki, Japan; 11 Department of Primary Care and Emergency Medicine, Graduate School of Medicine, Kyoto University, Kyoto, Japan; 12 Department of Preventive Services, Graduate School of Medicine and Public Health, Kyoto University, Kyoto, Japan

**Keywords:** Risk factors, Tomography, Respiratory distress syndrome

## Abstract

The best available evidence and the predictive value of computed tomography (CT) findings for prognosis in patients with acute respiratory distress syndrome (ARDS) are unknown. We systematically searched three electronic databases (MEDLINE, CENTRAL, and ClinicalTrials.gov). A total of 410 patients from six observational studies were included in this systematic review. Of these, 143 patients (34.9%) died due to ARDS in short-term. As for CT grade, the CTs used ranged from 4- to 320-row. The index test included diffuse attenuations in one study, affected lung in one study, well-aerated lung region/predicted total lung capacity in one study, CT score in one study and high-resolution CT score in two studies. Considering the CT findings, pooled sensitivity, specificity, positive likelihood ratio, negative likelihood ratio, and diagnostic odds ratio were 62% (95% confidence interval [CI] 30–88%), 76% (95% CI 57–89%), 2.58 (95% CI 2.05–2.73), 0.50 (95% CI 0.21–0.79), and 5.16 (95% CI 2.59–3.46), respectively. This systematic review revealed that there were major differences in the definitions of CT findings, and that the integration of CT findings might not be adequate for predicting short-term mortality in ARDS. Standardisation of CT findings and accumulation of further studies by CT with unified standards are warranted.

## Introduction

Acute respiratory distress syndrome (ARDS) is a respiratory failure disorder characterised by the rapid onset of widespread inflammation in the lungs^1^. The mortality rate of ARDS is as high as 40%^2,3^. Many studies have been conducted to identify predictors of acute illness; these predictors include age greater than 70 years, severity of illness scoring, cirrhosis, and sepsis^4–6^. However, no single factor was proven to be superior to the others.

We hypothesised that certain findings on computed tomography (CT) may be useful to accurately predict mortality. CT imaging is beneficial for the diagnosis of respiratory failure; bilateral opacities on chest CT are used as one of the diagnostic criteria in the Berlin definition^1^. CT has reportedly been more accurate than chest radiography in detecting the underlying causes and complications of ARDS^7^. Furthermore, several investigators have revealed that CT findings could predict mortality in ARDS^8–11^. For example, extensive opacities^12–14^, traction bronchiectasis^13,15^ and semi-quantitative score of several CT findings^8,15^ have been reported as possible poor prognostic factors. However, to the best of our knowledge, no systematic review of the predictive value of chest CT has been reported previously. Whether chest CT is beneficial for prognosis is an urgent clinical question in the management of ARDS.

To resolve this clinical question, we conducted a systematic review aimed to determine what types of CT findings were investigated and whether CT findings were predictive of short-term mortality in patients with ARDS.

## Methods

### Systematic review protocol

A systematic review and meta-analysis of the studies on diagnostic test accuracy (DTA) were conducted. We followed the methodological standards outlined in the *Handbook for DTA Reviews* of Cochrane^16^ and used the Preferred Reporting Items for a Systematic Review and Meta-analysis of DTA Studies^17^ to report our findings. The review protocol was prospectively registered with the University Hospital Medical Information Network Clinical Trials Registry (UMIN000040725). The need for ethical approval and consent was waived for this systematic review.

### Population, index test, and target condition

The target participants were patients with ARDS. We applied the definition of ARDS used in each study in order to collect the relevant studies comprehensively, including those that were published before the Berlin definition was published^1^. The index tests of interest were all findings on CT, defined in the primary studies. In this study, the target condition to be predicted was short-term mortality, and the reference standards of the condition were defined as 28-day mortality, 30-day mortality, 60-day mortality, or in-hospital mortality, along with the criteria defined by the primary study authors. This is because that The Guidelines on the management of ARDS by the British Thoracic Society define 28-day (almost equal to 30-day) mortality and in-hospital mortality as critically important indicators^18^ and that several clinical studies^19–21^ and a meta-analysis^22^ use 60-day mortality as a benchmark.

### Eligibility and study selection

We included all the studies, such as prospective, retrospective, and observational (cohort or cross-sectional) studies and secondary analyses of randomised controlled trial data, that investigated CT findings in patients with ARDS. We excluded case–control studies (two-gate study) and case studies that lacked DTA data, namely true positive (TP), false positive (FP), true negative (TN), and false negative (FN) values. Two authors independently screened each study for eligibility and extracted the data. Disagreements among reviewers were resolved via discussion or by a third reviewer.

### Electronic searching

To identify all eligible studies, we searched the Medical Literature Analysis and Retrieval System Online via PubMed, Cochrane Central Register of Controlled Trials (accessed on May 30th, 2020), and ClinicalTrials.gov. We restricted the literature to articles published in English. The details of the search strategy are described in the Supplementary File (Supplementary Table S1 and S2).

### Data extraction and quality assessment

The following data were extracted using a predefined data extraction form: study characteristics (author, year of publication, country, design, sample size, clinical settings, conflict of interest, and funding source), patient characteristics (inclusion/exclusion criteria and patient clinical and demographic characteristics), index test (computed tomography), reference standards (30-day mortality, 60-day mortality, or in-hospital mortality), and diagnostic accuracy parameters (TP, FP, FN, and TN). Two investigators evaluated the risk of bias using the Quality Assessment of Diagnostic Accuracy Studies 2 (QUADAS-2 tool), which included four risks of bias domains and three domains of applicability^23^. Any disagreements were resolved via discussion or by a third reviewer. Assessment findings were presented using a traffic light plot and a summary plot. Given the absence of evidence for publication bias in DTA studies and the lack of reliable methods for its assessment, no statistical evaluation of publication bias was performed^16^.

### Statistical analysis and data synthesis

For a predefined meta-analysis of all CT findings, the Cochrane Handbook for Systematic Reviews of DTA methodology was applied^16^. Diagnostic sensitivity and specificity estimates with 95% confidence intervals (CIs) were captured in paired forest plots to inspect the between-study variance. We used the hierarchical summary receiver operating characteristic (HSROC) random-effects model for meta-analysis. The HSROC model makes it possible to pool information across studies and derive smoothed estimates of covariate effects, components of variance, and individual study quantities^24^. In addition, the HSROC model accommodates the variations in cutoff values between studies. The pooled sensitivity and specificity with 95% CI were estimated at a fixed specificity as the median value of primary studies in the same manner as the previous Cochrane review and other systematic reviews^25–27^. All analyses were performed using Review Manager 5.4.1 (Cochrane Collaboration, London, United Kingdom), R version.3.5.3., Meta-DTA (Diagnostic Test Accuracy Meta-Analysis) application^28^ and CAST-HSROC (calculator for the summary points from the HSROC model) application^25^.

### Ethics statement

This study does not involve human participants.

## Results

### Study characteristics

**Figure 1:**
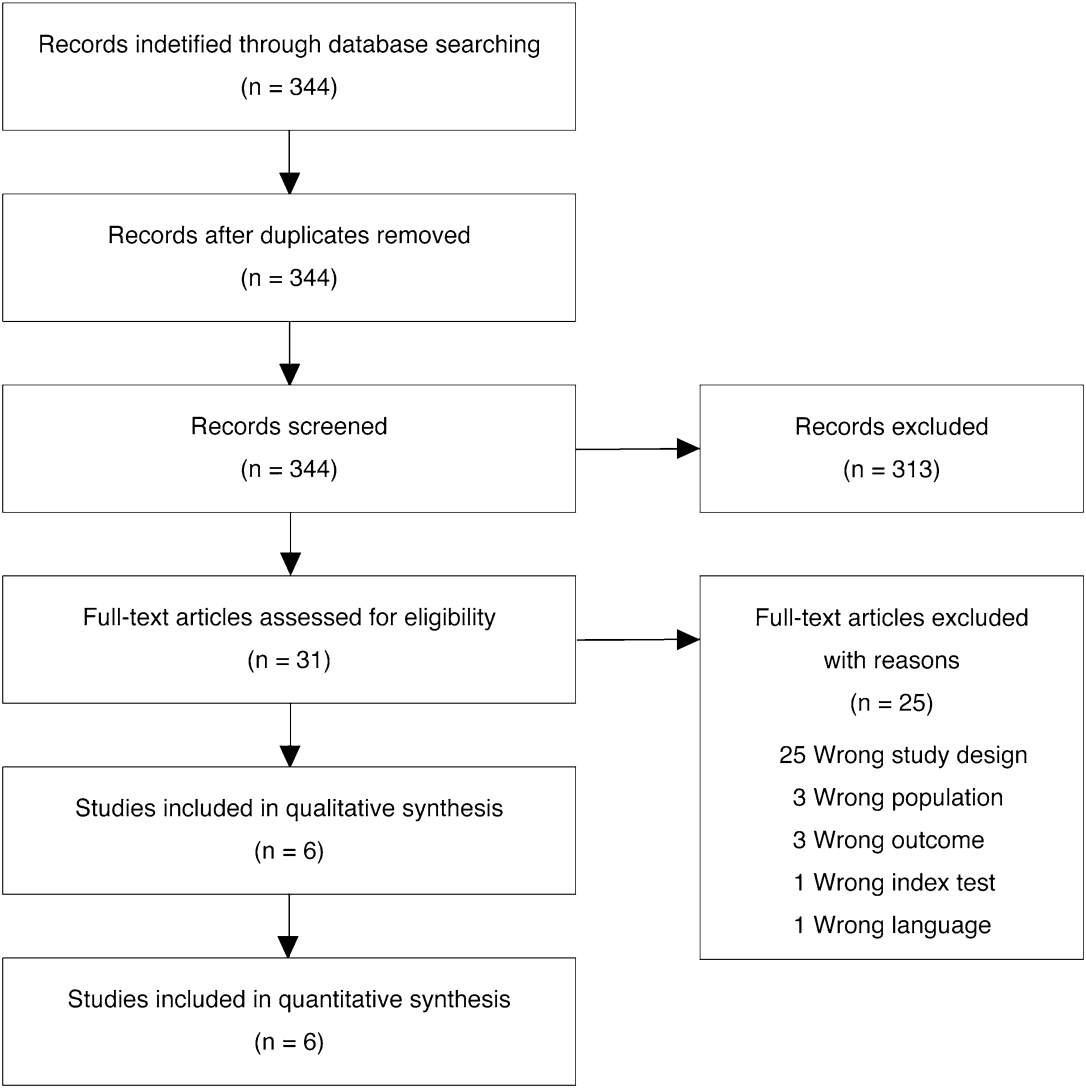
Flow diagram of the study selection.

Initially, 344 studies were screened. Six studies met the eligibility criteria and were included in the quality assessment and meta-analysis (Fig. 1) (Supplementary Table S3). A total of 410 patients from six observational studies were included (Table 1). Death due to ARDS in the short term occurred in 143 patients (34.9%). The median prevalence of mortality was 38.7% (interquartile range 24.5–49.5%). Two of the six studies were prospective in nature. Most studies (five of six studies) were conducted in the intensive care unit setting. Patient characteristics, index test definitions, and reference standards used in each study are summarized in Table 1.

**Table 1:** Summary of the primary study characteristics.

Author	Nishiyama	Kamo	Ichikado	Chung	Ichikado	Rouby
Year	2020	2019	2012	2011	2006	2000
Country	Japan	Japan	Japan	USA	Japan	France
Settings	ICU in a university hospital	ICU in a university hospital	ICU in a university hospital	General hospital	ICU in a university hospital	ICU in a university hospital
Number of patients	42	140	85	28	44	71
Age (mean, years)	64.2 ± 17.1	67 ± 17	75 ± 10	57.5 ± 15.0	61.8 ± 15.6	56 ± 17
Data collection	Retrospective	Retrospective	Prospective	Retrospective	Retrospective	Prospective
Enrolled period	2011 to 2013	2012 to 2015	2004 to 2008	1998 to 2006	2001 to 2002	1993 to 1997
Definition of ARDS	The Berlin definition	The Berlin definition	The AECC criteria	Histopathological diagnosis of DAD	The AECC criteria	The AECC criteria
**Severity**						
Index	P/F ratio	The Berlin definition	P/F ratio	–	Lung injury score	Lung injury score
Score	125.1 ± 57.7	Mild: 42 Moderate: 71 Severe: 40	96.2 ± 45.6	–	3.5 ± 0.7	2.9 ± 0.6
**Gas exchange**						
P_a_O_2_ (mean, Torr)	–	–	–	–	–	Lobar attenuations: 110 ± 39 Diffuse attenuations: 76 ± 42 Patchy attenuations: 82 ± 30
P_a_CO_2_ (mean, Torr)	–	Mild: 41.0 ± 9.6 Moderate: 47.4 ± 18.8 Severe: 46.7 ± 15.7	–	–	–	Lobar attenuations: 42 ± 6 Diffuse attenuations: 49 ± 11 Patchy attenuations: 47 ± 8
Aetiology of ARDS (%)	Aspiration (23.8%), Pneumonia (21.4%), Sepsis (16.7%), Surgery (11.9%), Trauma (4.8%), Others (21.4%)	Pneumonia (37%), Aspiration (28%), Sepsis (6.5%)	Pneumonia (37.6%), Sepsis (28.2%), Pulmonary (12.9%), Extrapulmonary (15.2%), Others (25.9)	Pneumonia (28.6%), Sepsis (10.7%), Aspiration (7.1%), Pancreatitis (7.1%), Drug reaction (7.1%), Recent major surgery (7.1%)	Pneumonia (36%), Sepsis (16%), Aspiration (7%), Postoperative (7%), Drug related (7%), Near drowning (5%), Pancreatitis (2%), Unknown (20%)	Primary ARDS (69.0%), Secondary ARDS (28.2%), ARDS of both origins (2.8%)
**Index test**						
CT modality	64-Row MDCT	320-Row MDCT	Various CT/MDCT systems	Various CT/MDCT systems	Various CT/MDCT systems	4-Row MDCT
CT findings	Well-aerated lung region/pTLC	HRCT score	HRCT score	Affected lung	CT score	Diffuse attenuations
Positive cutoff value	< 40%	> 210	> 210	> 80%	> 230	–
Timing of imaging	At diagnosis	Within 48 h of diagnosis	At diagnosis	Within 14 days of histopathological diagnosis	Within 7 days of diagnosis	–
Reference standard	30-Day mortality	30-Day mortality	60-Day mortality	In-hospital mortality	In-hospital mortality	In-hospital mortality

*AECC* American–European Consensus Conference, *ARDS* acute respiratory distress syndrome, *CT* computed tomography, *DAD* diffuse alveolar damage, *HRCT* high-resolution computed tomography, *ICU* intensive care unit, *MDCT* multi-detector computed tomography, *pTLC* predicted total lung capacity, *P*_*a*_*O*_*2*_ partial pressure of arterial oxygen, *P*_*a*_*CO*_*2*_ partial pressure of arterial carbon dioxide.

The index test was as follows: diffuse attenuations in one study^12^, affected lung in one study^13^, well-aerated lung region/predicted total lung capacity (pTLC) in one study^14^, CT score in one study^9^ and high-resolution CT (HRCT) score in two studies^8,15^. The CT findings of Rouby’s study were classified as diffuse, lobar, and patchy attenuations according to the extent and location of ground-glass opacity (GGO) and consolidation. The CT findings of Nishiyama’s study were classified as well-, poorly-, and non-aerated lung volume according to the Hounsfield units. In Chung’s study, GGO, consolidation, reticular opacities, traction bronchiectasis, and honeycombing were investigated. In studies by Ichikado and Kamo, CT and HRCT scores comprised all the six components of CT findings of normal attenuation, GGO, consolidation, GGO with traction bronchiectasis, consolidation with traction bronchiectasis, and honeycombing. Two different cutoff values have been reported across studies for the HRCT score (> 210 or 230). The definitions of each index test are provided in Supplementary Table S4. The spatial resolution of the CTs used in these studies differed greatly, ranging from 4-row to 320-row CTs.

### Risk of bias assessment

**Figure 2:**
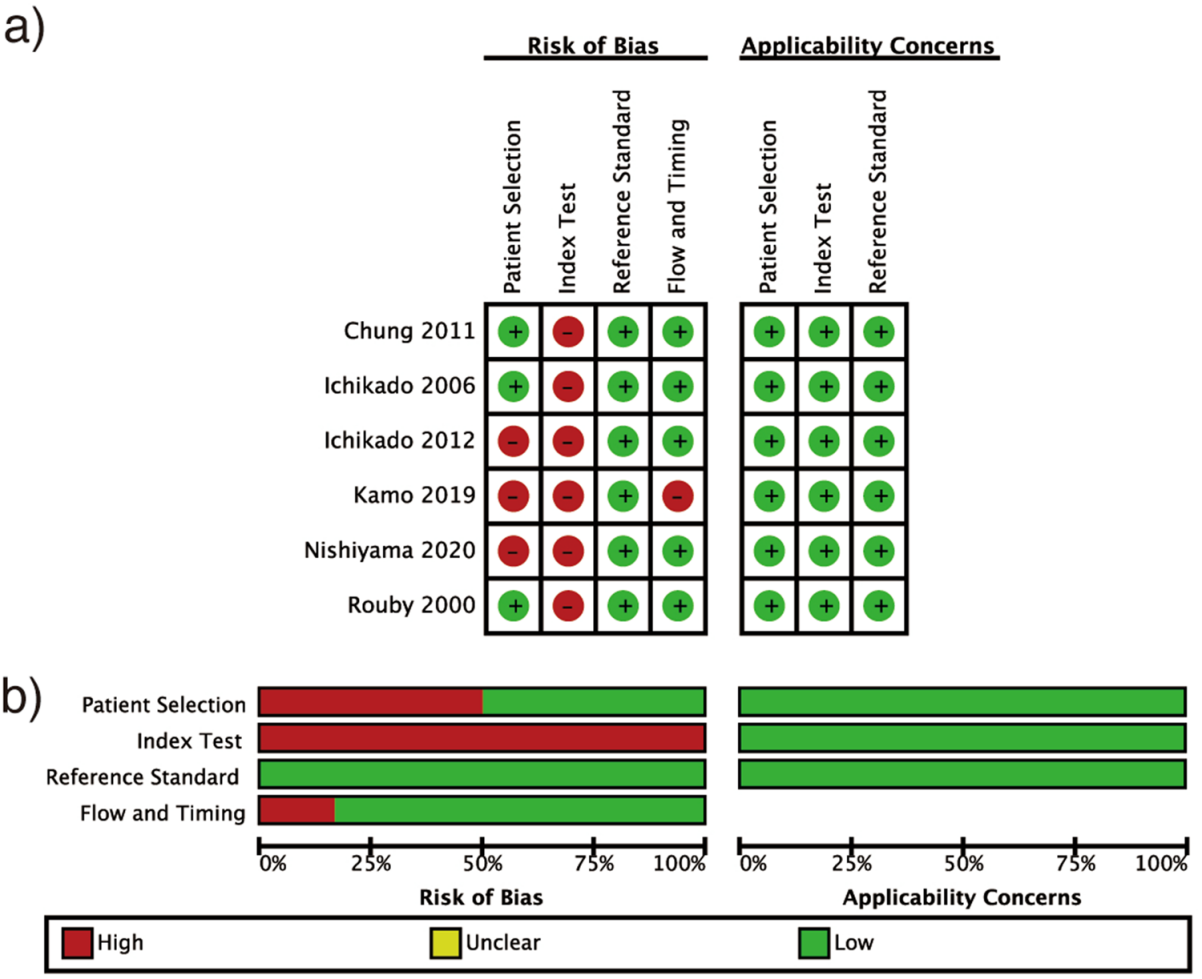
Risk of bias and applicability concerns (**a**) summary and (**b**) graph.

Based on patient selection, we considered three studies as having a high risk of bias (Fig. 2) (Supplementary Table S5) due to inappropriate exclusion criteria: emphysema, pregnancy, and patients without laboratory data were excluded in one study; while patients resuscitated from cardiopulmonary arrest were excluded in the remaining two studies. Considering the index test, we presumed all the studies to be at high risk since the reference standards were not blinded when the index tests were evaluated in four studies, and two studies did not define the test cutoff point previously. For the reference standard, we considered that no study had a high risk of bias or that there were no serious concerns regarding applicability as mortality seemed to be an objective fact and had to be evaluated accurately. In patient flow assessment, we assessed one study as having a high risk of bias because not all patients were included in the analysis. The overall risk of bias among the included studies was high.

Conversely, there were no serious concerns regarding the applicability of the studies.

### Meta-analysis and predictive value

**Figure 3:**
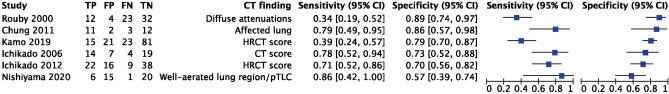
Paired forest plot. TP, true positive; FP, false positive; TN, true negative; FN, false negative; CT, computed tomography; HRCT, high resolution computed tomography; pTLC, predicted total lung capacity; CI, confidence interval.

**Figure 4:**
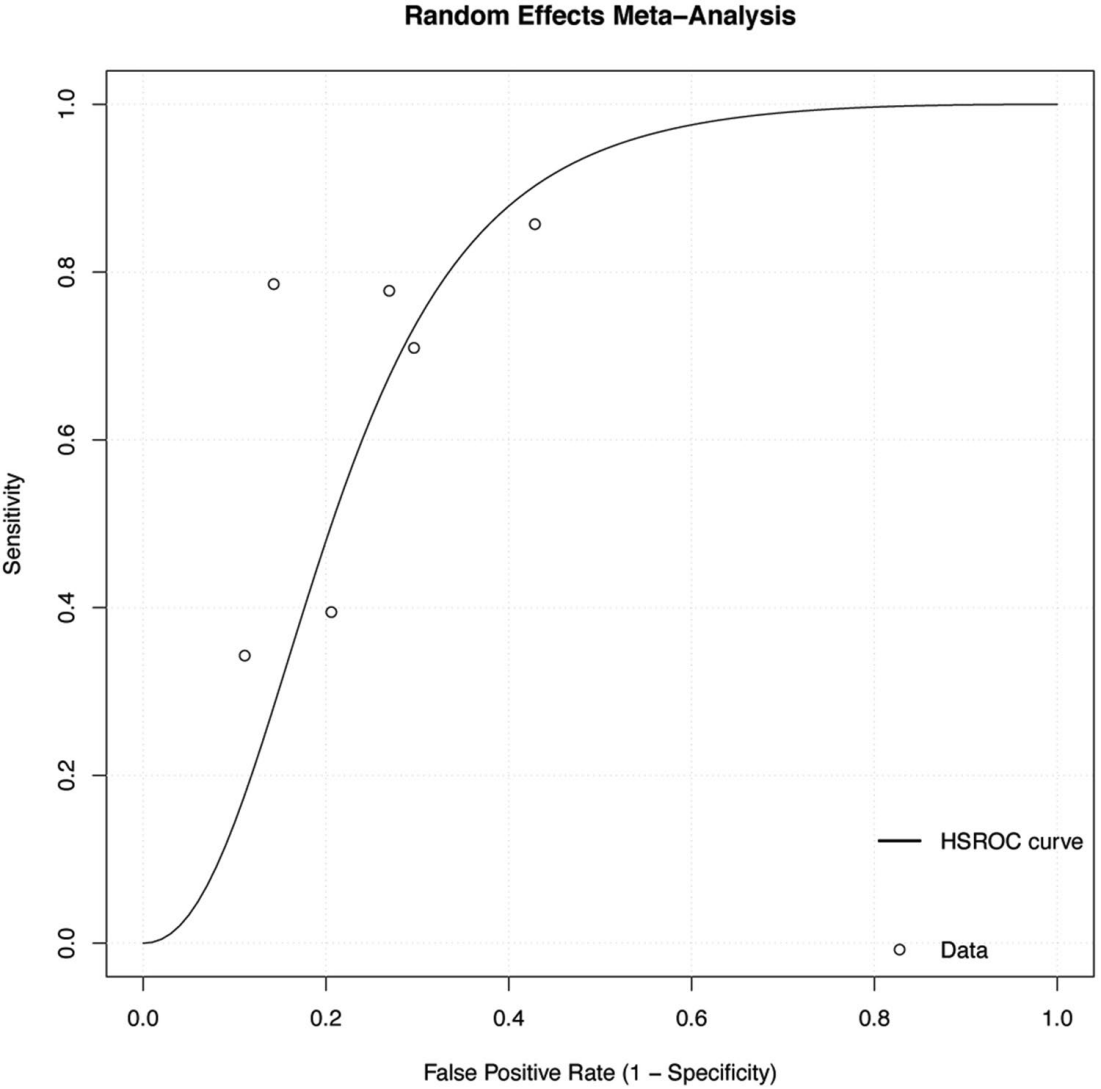
HSROC curve. HSROC, hierarchical summary receiver operating characteristic.

The differences in the index tests were found to be high. However, since the predefined protocol stipulated that a meta-analysis be performed, we tentatively performed the analysis. The predictive value of CT findings in each study is presented as a forest plot in Fig. 3. Using the HSROC model, a summary ROC curve was plotted (Fig. 4) (Supplementary Table S6). At a fixed specificity of 76% as the median value of the primary study, the pooled sensitivity was 62% (95% CI 30–88%). At this point, the positive likelihood ratio, negative likelihood ratio, and diagnostic odds ratio were 2.58 (95% CI 2.05–2.73), 0.50 (95% CI 0.21–0.79), and 5.16 (95% CI 2.59–3.46), respectively (Supplementary Fig. S1).

## Discussion

This systematic review of six studies revealed that CT findings greatly differed in patients with ARDS. As for CT modality, the CTs used ranged from 4- to 320-row, and the CT findings investigated were GGO, consolidation, reticular shadows, traction bronchiectasis, honeycomb lung, or their integration. Tentative meta-analysis showed low sensitivity and specificity for predicting short-term mortality in patients with ARDS (pooled sensitivity 62% [95% CI 30–88%], pooled specificity 76% [95% CI 57–89%]). Both pooled sensitivity and specificity had wide 95% CIs.

We have identified three key strengths of this study. To the best of our knowledge, this is the first systematic review to investigate the prognostic ability of CT for predicting mortality in ARDS. CT is widely used in advanced medical institutions worldwide, and specific CT findings are used as diagnostic criteria for ARDS^1^. However, CT also has certain disadvantages, such as the manpower required to transport patients, patient safety concerns^29^, the economic cost of CT imaging, and high dose of ionising radiation exposure^30–33^. Thus, CT imaging should be performed based on the evidence of clinical utility. This review has demonstrated that the study of CT findings and prognosis is an unexplored field and has potential for future development. Second, we focused on the specific CT findings, including diffuse attenuations in one study, affected lung in one study, well-aerated lung region/pTLC in one study, CT score in one study and HRCT score in two studies. There is no essential difference in the measurement methods between HRCT score and CT score, but caution should be paid to the fact that the cutoff values for the index tests are different (> 210 or 230). On the other hand, since the Ichikado’s study (2012)^8^ and the Kamo’s study (2019)^15^ used the same name, the same measurement method, and the same cutoff value, we considered it acceptable to judge them as the same index test. All the findings were based on GGO, consolidation, honeycombing, traction bronchiectasis, intralobular septal wall thickening, change of Hounsfield units, distribution of opacity, or their combination (Supplementary Table S4, Fig. S2). However, there is no established consensus regarding the specific CT findings that should be the focus of the management of ARDS. Third, this study was conducted in accordance with the Cochrane Handbook for Systematic Reviews of DTA. Previous systematic reviews of prognostic factors in ARDS have included pathological examination by open lung biopsy^34^, extravascular lung water index^35^, and various serum biomarkers (C-reactive protein, cytokines, N-terminal pro-brain natriuretic peptide, and circulating angiopoietin-2)^36–38^. Nevertheless, none of these studies have been reviewed in a manner consistent with the principles of the DTA Handbook. A systematic review of DTA should be considered separately from a systematic review of interventions^39–41^. This is because DTA reviews use their own indices, such as index test, reference standard, and target condition and use specific evaluation methods, such as the QUADAS-2 tool for bias evaluation^23,42^. Our method could provide a methodological basis for future diagnostic and prognostic studies of ARDS.

The results of this meta-analysis demonstrated that the integration of CT findings might not be a reliable prognostic tool for patients with ARDS. This is because CT has several disadvantages for predicting mortality: 1) timing of imaging, 2) quality of images, and 3) causes of death in patients with ARDS. The timing of CT imaging plays an important role in mortality prediction. Generally, ARDS images show various patterns depending on disease progression. Typical CT findings in ARDS include extensive consolidation/GGOs in the acute phase and fibrotic changes (e.g., traction bronchiectasis or honeycomb lung) in the late phase^43,44^. These changes in the CT findings do not progress homogeneously, and CT findings can also be affected by therapeutic interventions. For instance, fluid management^45,46^, drugs^47^, and respiratory settings including lung protective ventilation^48–50^, recruitment manoeuvers^51,52^, and prone position ventilation^53^. Therefore, it remains controversial whether CT imaging is the most appropriate tool for the predicting prognosis in patients with ARDS in clinical practice. This review shows that the timing of imaging was not standardised in each study (Table 1), which may have resulted in inappropriate timing of imaging for predicting prognosis. Further, CT image quality is an issue. In current practice, multiple detector CT (MDCT) is the usual imaging technology. Even between MDCTs, a tenfold difference in special resolution has been reported (slice thickness in 4-row CT, 5.0 mm; slice thickness in 320-row CT, 0.5 mm)^54–56^. In this primary study, the number of detector rows included covered a wide range, from 4- to 320-rows (Table 1). Low-quality CT could miss important findings, such as GGO or traction bronchiectasis. The presence of GGO is a well-known indicator of early fibrosis^57–60^. To avoid missing these findings, it would be necessary to use high-quality CT whenever possible. In addition to the previous two restrictions, the cause of death in ARDS is disadvantageous for CT. It has been pointed out that the severity of lung injury may not correlate with mortality. A prospective observational study found that there was no difference in 28-day mortality between mild and moderate ARDS according to the Berlin definition (mild, 30.9%; moderate, 27.9%; *p* = 0.70)^61^. According to previous studies, the most common cause of death in ARDS was multiple organ failure, accounting for 30–50% of deaths^62,63^. The mortality rate increases with the number of failing organs other than the lungs^63^. It has been reported that respiratory failure accounted for only 13–19% of all ARDS deaths^62,63^; it could be difficult to predict prognosis based on the severity of pulmonary injury on CT alone. Our results suggest that attention should be paid to organs other than the lungs to accurately estimate prognosis in patients with ARDS.

This study has several limitations. First, there were a limited number of studies and some retrospective studies were included in this study, which could cause a type-2 error. Pooled sensitivity and specificity had wide CIs; therefore, caution is required when applying these findings to clinical practice. Second, there was some heterogeneity among the included studies. The definitions of the index tests were not homogeneous, and the cutoff points differed even among studies assessing the extent of lung damage. The definition of ARDS was not common across studies, and there was heterogeneity among the patients. It is important to enrol patients using the Berlin definition and standardise the definition of CT findings in future studies. Third, the designs of the studies included in this review were not suitable for assessing predictive value. Because we assumed that few studies had evaluated the predictive value of CT findings in patients with ARDS, we planned to include descriptive and exploratory studies. Extensive inclusion criteria may have reduced the quality of the included studies. Fourth, there was a high risk of bias in all studies, which may have affected the estimates. Most studies did not specify index test thresholds a priori, and the index test results were interpreted without blinding the reference standard results. These biases could be partially attributed to the study design. Additional studies with predefined CT findings are required. Finally, there were no patients with ARDS due to severe acute respiratory syndrome coronavirus 2 (SARS-CoV-2) infection, even though this review was conducted during the SARS-CoV-2 epidemic. Further caution should be applied when evaluating CT findings in patients with ARDS due to SARS-CoV-2 infection.

In conclusion, patients with ARDS present with various CT findings. The evaluation of CT findings was not standardised in previous studies. This systematic review revealed that the integration of CT findings might not be adequate for predicting short-term mortality in patients with ARDS. Standardisation of CT findings and the accumulation of further studies by CT with unified standards are warranted.

## Supplementary Information

Supplementary Information.

## Data Availability

No additional data available.
