# Supplementary material for: Predictive value of computed tomography for short-term mortality in patients with acute respiratory distress syndrome: a systematic review
Source: Sci Rep. 2022 Jun 10;12:9579. doi: 10.1038/s41598-022-13972-x (PMC9185136; doi:10.1038/s41598-022-13972-x)
Supplement: Supplementary file 1 — Supplementary Information. [file 41598_2022_13972_MOESM1_ESM.docx]

**Supplementary Information**

**Contents**

| **Supplementary Table S1. Search strategy for Medical Literature Analysis and Retrieval System Online (MEDLINE)** |
| --- |
| **Supplementary Table S2. Search strategy for Cochrane Central Register of Controlled Trials** |
| Supplementary Table S3. Excluded literature by full-text screening |
| Supplementary Table S4. Definitions of each index test |
| Supplementary Table S5. Details of the assessment of QUDAS-2 tool |
| Supplementary Table S6. HSROC parameters |
| Supplementary Figure S1. Calculator for the summary point from the HSROC Model |
| Supplementary Figure S2. Typical high-resolution CT findings in patients with ARDS |

* The review protocol was prospectively registered with the University Hospital Medical Information Network Clinical Trials Registry (UMIN000040725).

**Supplementary Table S1. Search strategy for Medical Literature Analysis and Retrieval System Online (MEDLINE)**

| **Search number** | **Query** | **Results** |
| --- | --- | --- |
| #1 | Respiratory Distress Syndrome, Adult [mh] | 19,356 |
| #2 | Acute lung injury [mh] | 6,231 |
| #3 | ALI [tiab] OR ARDS [tiab] | 18,076 |
| #4 | Acute lung injur* [tiab] OR Acute respiratory distress [tiab] OR Acute respiratory failure[tiab] | 31,624 |
| #5 | "severe respiratory failure"[tiab] | 1,418 |
| #6 | #1 OR #2 OR #3 OR #4 OR #5 | 47,232 |
| #7 | (High Resolution Computed Tomography[tiab] OR HRCT[tiab] OR high-resolution CT[tiab] OR tomography, x-ray computed[mh]) AND lung[mh] | 15,632 |
| #8 | #6 AND #7 | 426 |
| #9 | animals[mh] NOT humans[mh] | 4,709,914 |
| #10 | #8 NOT #9 | 343 |

**Supplementary Table S2. Search strategy for Cochrane Central Register of Controlled Trials**

| **ID** | **Search** | **Results** |
| --- | --- | --- |
| #1 | [mh "Respiratory Distress Syndrome, Adult"] | 1302 |
| #2 | [mh "Acute lung injury"] | 440 |
| #3 | ALI:ti,ab OR ARDS:ti,ab | 3217 |
| #4 | "Acute lung injury":ti,ab OR "Acute respiratory distress":ti,ab OR "Acute respiratory failure":ti,ab | 2933 |
| #5 | "severe respiratory failure":ti,ab | 121 |
| #6 | {OR #1-#5} | 5388 |
| #7 | ("High Resolution Computed Tomography":ti,ab OR HRCT:ti,ab OR "high-resolution CT":ti,ab OR [mh "tomography, x-ray computed"]) AND [mh lung] | 40 |
| #8 | #6 AND #7 | 1 |
| #9 | [mh animals] NOT [mh humans] | 6792 |
| #10 | #8 NOT #9 | 1 |

**Supplementary Table S3. Excluded literature by full-text screening**

| **Title** | **Year** | **Author** | **Reason** |
| --- | --- | --- | --- |
| Radiology in the diagnosis of adult respiratory distress syndrome with special reference to pulmonary oedema--its distribution and detection | 1988 | Wegenius, G | Wrong study design |
| Understanding images: correlation between computerized tomographic scans of lung structure with impaired function in ARDS | 1988 | Zapol, W M | Wrong study design |
| CT scan in ARDS: clinical and physiopathological insights | 1991 | Gattinoni, L | Wrong study design |
| Correlative study of adult respiratory distress syndrome by light, scanning, and transmission electron microscopy | 1992 | Anderson, W R | Wrong study design or index test |
| CT and chest radiography in the evaluation of adult respiratory distress syndrome | 1994 | Tagliabue, M | Wrong study design or population |
| Computed tomography in established adult respiratory distress syndrome. Correlation with lung injury score | 1994 | Owens, C M | Wrong study design |
| Acute respiratory distress syndrome: CT abnormalities at long-term follow-up | 1999 | Desai, S R | Wrong study design |
| Regional distribution of gas and tissue in acute respiratory distress syndrome. I. Consequences for lung morphology. CT Scan ARDS Study Group | 2000 | Puybasset, L; Cluzel | Wrong study design or outcome |
| Regional distribution of gas and tissue in acute respiratory distress syndrome. III. Consequences for the effects of positive end-expiratory pressure. CT Scan ARDS Study Group. Adult Respiratory Distress Syndrome | 2000 | Puybasset, L; Gusman | Wrong study design |
| Acute respiratory distress syndrome caused by pulmonary and extrapulmonary injury: a comparative CT study | 2001 | Desai, S R | Wrong study design |
| Computerised tomography scan imaging in acute respiratory distress syndrome | 2001 | Pesenti, A | Wrong study design |
| Can the tomographic aspect characteristics of patients presenting with acute respiratory distress syndrome predict improvement in oxygenation-related response to the prone position? | 2002 | Papazian, Laurent | Wrong study design |
| Measurement of alveolar derecruitment in patients with acute lung injury: computerized tomography versus pressure-volume curve | 2006 | Lu, Qin | Wrong study design |
| Radiological imaging in acute lung injury and acute respiratory distress syndrome | 2006 | Caironi, Pietro | Wrong study design |
| Causes and prognosis of diffuse alveolar damage diagnosed on surgical lung biopsy | 2007 | Parambil, Joseph G | Wrong study design |
| Qualitative and quantitative CT analysis of acute pulmonary failure | 2009 | Reske, A W | Wrong study design or language |
| Diagnostic contribution from pulmonary biopsies in hematology patients with acute respiratory failure from undetermined etiology | 2013 | Gay, J | Wrong study design or population |
| Lung inhomogeneity in patients with acute respiratory distress syndrome | 2014 | Cressoni, Massimo | Wrong study design |
| Detection of fibroproliferation by chest high-resolution CT scan in resolving ARDS | 2014 | Burnham, Ellen L | Wrong study design or outcome |
| A follow-up study on acute respiratory distress syndrome survivors after extracorporeal membrane oxygenation by pulmonary high-resolution CT | 2015 | Li, Xu-Yan | Wrong study design |
| Lung inhomogeneities, inflation and [18F]2-fluoro-2-deoxy-D-glucose uptake rate in acute respiratory distress syndrome | 2016 | Cressoni, Massimo | Wrong study design or outcome |
| Lung Recruitment Assessed by Respiratory Mechanics and Computed Tomography in Patients with Acute Respiratory Distress Syndrome. What Is the Relationship? | 2016 | Chiumello, Davide | Wrong study design |
| The clinical benefit of a follow-up thoracic computed tomography scan regarding parenchymal lung injury and acute respiratory distress syndrome in polytraumatized patients | 2017 | Negrin, Lukas | Wrong study design or population |
| Clinical characteristics and prognosis of drug-associated acute respiratory distress syndrome compared with non-drug-associated acute respiratory distress syndrome: a single-centre retrospective study in Japan. | 2017 | Anan, Keisuke | Wrong study design |
| Automatic quantitative computed tomography segmentation and analysis of aerated lung volumes in acute respiratory distress syndrome-A comparative diagnostic study | 2017 | Klapsing, Philipp | Wrong study design |

Supplementary Table S4. Definitions of each index test

| **Author, Year** | **Index test** | **Definition** |
| --- | --- | --- |
| Nishiyama, 2020 [1] | Well-aerated lung region/pTLC | Lung regions were classified into 4 categories by CT attenuation densities: 1) hyperinflated, density between -1000 and -901 Hounsfield units (HU); 2) normally aerated, density between -900 and -501 HU; 3) poorly aerated, density between -500 and -101 HU; and 4) non-aerated, density between +100 and -100 HU. The volume of each lung region was divided by pTLC to correct for differences in physique among patients. We considered hyperinflated and normally aerated regions (density below -500 HU) as well-aerated lung regions for this study and defined the well-aerated group and less-aerated group based on the median percentage of well-aerated lung region/pTLC. |
| Kamo, 2019 [2] | HRCT score | HRCT findings were graded on a scale of 1–6 based on the classification system correlating with previously de- scribed pathology: 1, normal attenuation; 2, ground-glass attenuation; 3, consolidation; 4, ground-glass attenuation with traction bronchiolectasis or bronchiectasis; 5, consolidation with traction bronchiolectasis or bronchiectasis and 6, honeycombing. The presence of each of these six abnormalities was assessed independently in three (upper, middle and lower) zones of each lung. The abnormality score for each zone was calculated by multiplying the percentage area by the point value (1–6). The six zone scores were aver- aged to determine the total score for each abnormality in each patient. |
| Ichikado, 2012 [3] | HRCT score | HRCT findings were graded on a scale of 1–6 based on the classification system correlating with previously described pathology: 1, normal attenuation;2, ground-glass attenuation; 3, consolidation; 4, ground-glass attenuation with traction bronchiolectasis or bronchiectasis; 5, consolidation with traction bronchiolectasis or bronchiectasis and 6, honeycombing. The presence of each of these six abnormalities was assessed independently in three (upper, middle and lower) zones of each lung. The abnormality score for each zone was calculated by multiplying the percentage area by the point value (1–6). The six zone scores were aver- aged to determine the total score for each abnormality in each patient. |
| Chung, 2011 [4] | Affected lung | Percentage of lung involvement was estimated qualitatively to the nearest 10th percentile (e.g. 10%, 20%, etc.). Ground-glass opacities were defined as lung opacities that did not obscure blood vessels. Consolidation was defined as lung opacities that did obscure blood vessels. |
| Ichikado, 2006 [5] | CT score | The thin-section CT findings were graded on a scale of 1– 6 on the basis of the classification system previously de- scribed (15): score of 1, normal attenuation; score of 2, ground-glass attenuation; score of 3, consolidation; score of 4, ground-glass attenuation with traction bronchiolectasis or bronchiectasis; score of 5, consolidation with traction bronchiolectasis or bronchiectasis; and score of 6, honeycombing. The presence of each of these six abnormalities was assessed independently in three (upper, middle, and lower) zones of each lung. The ab- normality score for each zone was calculated by multiplying the percentage area by the point value (the score of 1–6). The six zone scores were aver- aged to determine the total score for each abnormality in each patient. |
| Rouby, 2000 [6] | Diffuse attenuations | Classification of the chest radiograph was as follows: “lobar” if hyperattenuations areas involved essentially the lower lobes; “diffuse” if hyperattenuated areas were equally disseminated within the upper and lower lobes, presenting the characteristic feature of “white lungs”; “patchy” if hyperattenuated areas involved the upper and lower lobes with a persistent aeration of a part of the upper lobes. |

HRCT, high-resolution computed tomography; pTLC, predicted total lung capacity

**Supplementary Table S5. Details of the assessment of QUDAS-2 tool**

|  |  | **Patients selection** | | | **Index test** | | | **Reference standard** | | **Patients flow** | |
| --- | --- | --- | --- | --- | --- | --- | --- | --- | --- | --- | --- |
| **Author** | **Year** | **Bias** | **Reason** | **Applicability** | **Bias** | **Reason** | **Applicability** | **Bias** | **Applicability** | **Bias** | **Reason** |
| Rouby, et al | 2000 | Low |  | Low concern | High | Not  pre-specified | Low concern | Low | Low concern | Low |  |
| Ichikado, et al | 2006 | Low |  | Low concern | High | Not  pre-specified | Low concern | Low | Low concern | Low |  |
| Chung, et al | 2011 | Low |  | Low concern | High | Blind was unclear | Low concern | Low | Low concern | Low |  |
| Ichikado, et al | 2012 | High | Inappropriate exclusion | Low concern | High | Blind was unclear | Low concern | Low | Low concern | Low |  |
| Kamo, et al | 2019 | High | Inappropriate exclusion | Low concern | High | Not pre-specified  Blind was unclear | Low concern | Low | Low concern | High | Inappropriate Exclusion in analysis |
| Nishiyama, et al | 2020 | High | Inappropriate exclusion | Low concern | High | Not pre-specified and not blind | Low concern | Low | Low concern | Low |  |

QUADAS, Quality Assessment of Diagnostic Accuracy Studies

**Supplementary Table S6. HSROC parameters**

| **Parameter name** | **Value** |
| --- | --- |
| θ | -0.569 |
| λ | 1.954 |
| β | -0.743 |
| σ_θ_ | 0.334 |
| σ_α_ | 0.000 |

HSROC, hierarchical summary receiver operating characteristic

**SupplementaryFigure** **S1**. **Calculator for the summary point from the HSROC Model**

A) Probability density plot


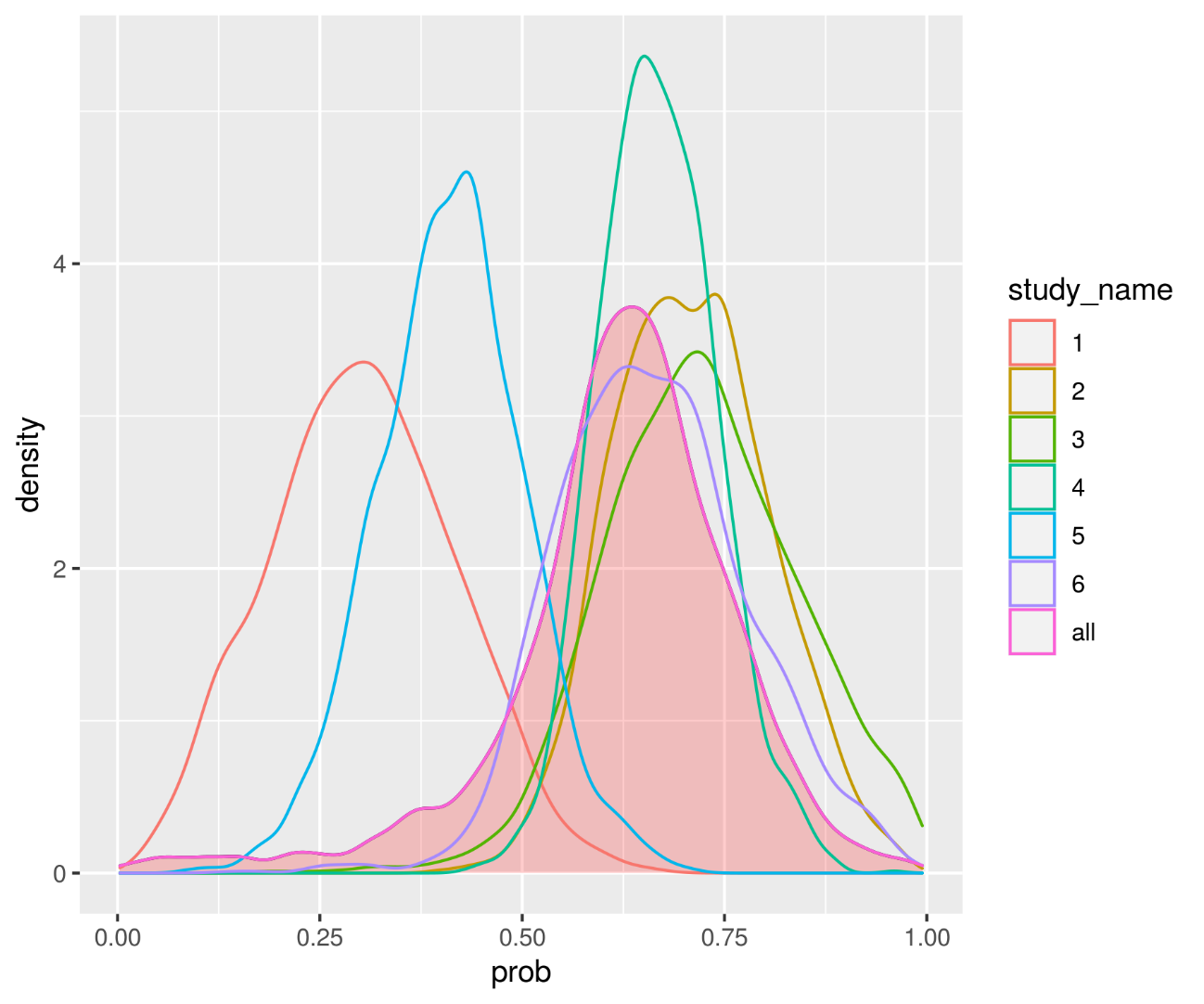


Study_name was followed as:

1, Rouby 2000; 2, Ichikado 2006; 3, Chung 2011; 4, Ichikado 2012; 5, Kamo 2019; 6 Nishiyama 202

^*^ The pooled sensitivity was calculated from the fixed specificity (0.760, as median of all studies).

b) Markov chain trace plot


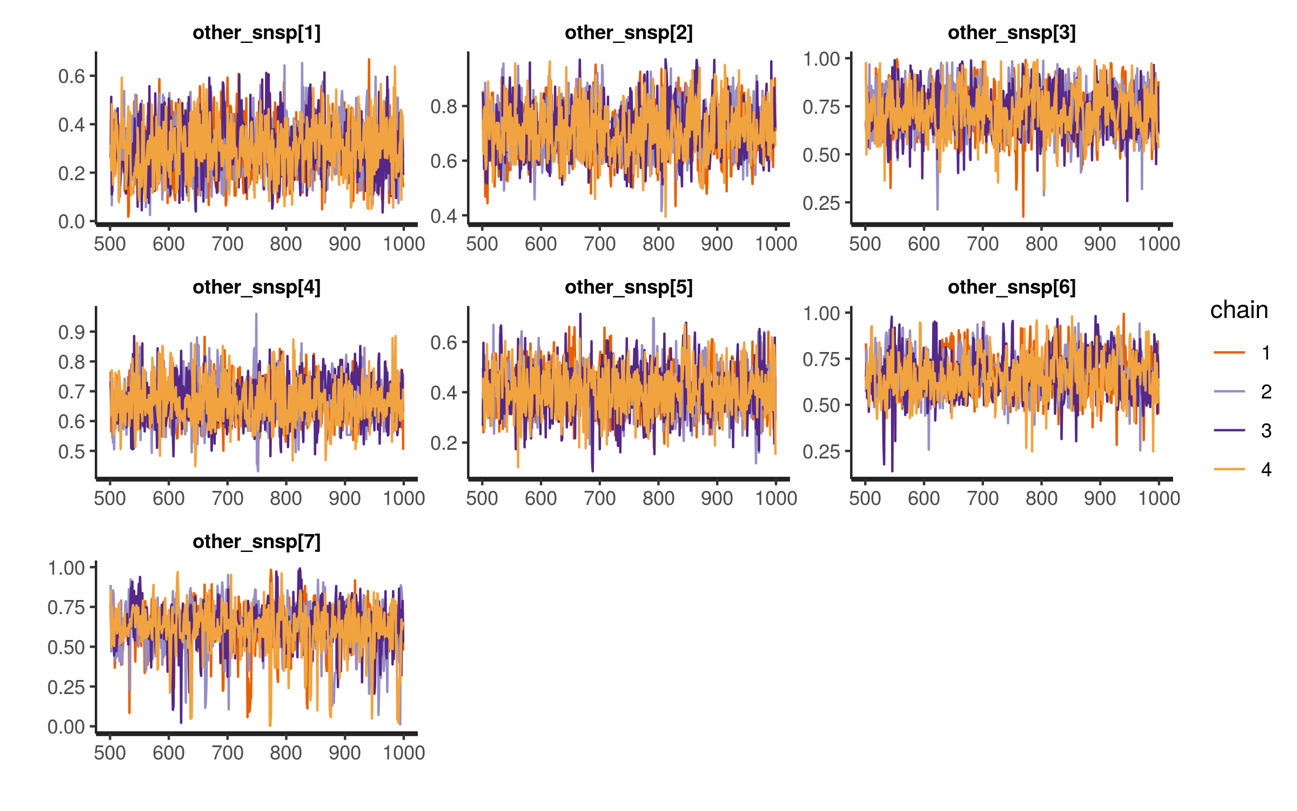


^*^ The pooled sensitivity was calculated from the fixed specificity (0.760, as median of all studies).

HSROC, hierarchical summary receiver operating characteristic

**
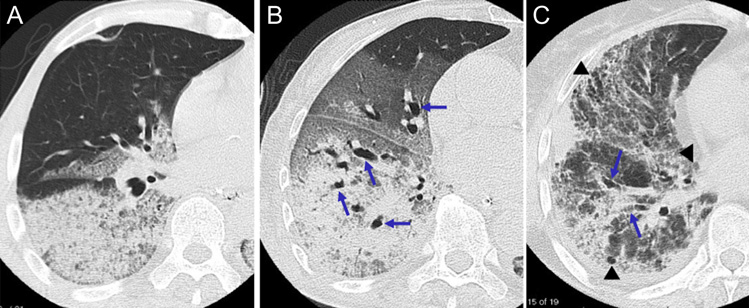
SupplementaryFigure S2. Typical high-resolution CT findings in patients with ARDS**

High-resolution CT (HRCT) findings correlated with pathology was cited by a previous study [3].

(A) HRCT findings corresponding to exudative phase of ARDS. HRCT scan at the level of right middle lobe shows dependent airspace consolidation without traction bronchiectasis and nondependent areas of sparing. The patient was a 68-year-old man with ARDS due to Streptococcus pneumonia. (B) HRCT findings corresponding to fibroproliferative phase of ARDS. HRCT scan at the level of right lower lobe shows extensive airspace consolidation and ground-glass attenuation associated with traction bronchiectasis (arrows). The patient was an 84-year-old woman with ARDS due to sepsis. (C) HRCT findings corresponding to fibrotic phase of ARDS. HRCT scan at the level of right inferior pulmonary vein shows extensive ground-glass attenuation associated with traction bronchiectasis (arrows), coarse reticulation and cystic changes (arrowheads). The patient was a 65-year-old woman with ARDS due to viral pneumonia.

1 Nishiyama, A. *et al.* A predictive factor for patients with acute respiratory distress syndrome: CT lung volumetry of the well-aerated region as an automated method. *Eur J Radiol.* **122**,108748(2020).

2 Kamo, T. *et al.* Prognostic values of the Berlin definition criteria, blood lactate level, and fibroproliferative changes on high-resolution computed tomography in ARDS patients. *BMC Pulm Med.* **19**,37(2019).

3 Ichikado, K. *et al.* Fibroproliferative changes on high-resolution CT in the acute respiratory distress syndrome predict mortality and ventilator dependency: a prospective observational cohort study. *BMJ Open.* **2**,e000545(2012).

4 Chung, J. H., Kradin, R. L., Greene, R. E., Shepard, J. A. & Digumarthy, S. R. CT predictors of mortality in pathology confirmed ARDS. *Eur Radiol.* **21**,730-737(2011).

5 Ichikado, K. *et al.* Prediction of prognosis for acute respiratory distress syndrome with thin-section CT: validation in 44 cases. *Radiology.* **238**,321-329(2006).

6 Rouby, J. J. *et al.* Regional distribution of gas and tissue in acute respiratory distress syndrome. II. Physiological correlations and definition of an ARDS Severity Score. CT Scan ARDS Study Group. *Intensive Care Med.* **26**,1046-1056(2000).
